# The effect of commercial functional food with probiotics on microorganisms from early carious lesions

**DOI:** 10.1038/s41598-020-67775-z

**Published:** 2020-07-01

**Authors:** María del Pilar Angarita-Díaz, Johanna C. Arias, Claudia Bedoya-Correa, María J. Cepeda, María F. Arboleda, Juan M. Chacón, Yenny Leal

**Affiliations:** 1 School of Dentistry, Universidad Cooperativa de Colombia, Carrera 22 # 7-06 Sur, Villavicencio, Colombia; 2 School of Dentistry, Universidad Cooperativa de Colombia, Medellín, Colombia

**Keywords:** Health care, Dentistry, Oral microbiology

## Abstract

Caries rates in school-age children are still high enough to be the cause of serious concern for health systems in different countries. The biotechnology strategies studied to decrease these rates include the consumption of probiotics—available via a variety of functional foods obtainable on the market—that are able to inhibit bacteria associated with this disease. In this vein, the purpose of this study was to determine the effect of these foods on the growth of microorganisms in early carious lesions in children aged between 6 and 12. In the first phase, an agar well diffusion method was applied to selected foods, available in supermarkets, which contain probiotics that have already been shown to inhibit *Streptococcus* *mutans* (ATCC 25175), and to lower the pH in liquid culture media. In a second phase, these foods (n = 4) were examined in terms of their ability to inhibit the microorganisms in contact with early carious lesions in children and to reduce the pH of mixed cultures combined with the food. The results revealed that, of the foods tested, three inhibit the growth of microorganisms in carious lesions and, at the same time, lower the pH of the culture by more than 2.5 units. The food with the highest inhibitory capacity (14 mm, IQR 13–14) showed a similar effect among patients (P > 0.05), which together with the fact that its sugar concentration is less than 10%, makes it an ideal candidate for clinical study.

## Introduction

Dental caries represent the most widespread non-communicable disease globally and, for this reason, constitute a public health problem. Despite the contribution of fluoride to reducing the rates of dental caries, the disease continues to have a high level of incidence from an early age^1^. A diet rich in fermentable carbohydrates is one of the reasons for this, as cariogenic microorganisms in dental biofilm can metabolize these carbohydrates very rapidly, producing the acids that demineralize teeth^2^. This is one of the reasons for which organizations such as the World Health Organization (WHO) recommend a daily sugar intake of less than 10% of total caloric intake, and even advise that a 5% intake would be even more beneficial to oral health^3^.

The consequences of dental caries can affect quality of life, given the acute and chronic medical conditions that they can cause, as well as the socioeconomic problems that contribute to inequality that affects a country’s development and, in turn, a population’s access to healthcare^4^. The first and second permanent molars erupt between the ages of 6–12. The former are most at risk for caries and, in fact, are the most frequently affected by them, given their retentive tooth morphology and the fact that they are difficult to see because of their location^5^. In Colombia, the last Estudio de Salud Bucal (ENSAB IV) (Oral Health Study) reported that 54.2% of 12-year-olds already had caries in their permanent teeth, a figure that motivates further research on strategies that could complement the role of fluoride in preventing this disease^6^.

One of these strategies involves the consumption of foods with components that inhibit or reduce the effects of cariogenic microorganisms. These include milk, cacao, some fruits such as grapes, and propolis among others, or functional foods defined as foods enriched with components that provide health benefits^7,8^.

These cover foods supplemented with probiotics, which are microorganisms, mainly bacteria, that are safe for human consumption, and that when ingested in sufficient quantities can be beneficial for human health^9^.

There is a wide variety of functional foods supplemented with probiotics on the market that improve the immune system’s response and enhance the functioning of the digestive system. The majority of these probiotic bacteria (*Lactobacillus* *rhamnosus* GG, *Lactobacillus* *reuteri*, *Lactobacillus* *casei*, *Lactobacillus* *brevis*, *Lactobacillus* *acidophilus*, *Bifidobacterium* *lactis*, *Bifidobacterium* *longum*) have also been studied in order to find their possible capacity to control oral cavity diseases such as caries. This is due to their known functions such as the inhibition of cariogenic microorganisms by means of the production of toxic substances and competition for nutrition and space, and by encouraging increased oral cavity immune responses^10–17^.

Given the information detailed above, it would be interesting to study whether there are functional foods supplemented with probiotics on the market that reduce cariogenic microorganisms and so be able to define candidates to be evaluated in clinical trials. If favorable results are found in such studies, they could determine specific foodstuffs that could contribute to children’s oral health programs. The purpose of this study was to analyze the effect of commercially-available functional foods supplemented with probiotics on the inhibitory and pH-lowering capacity of mixed microbial cultures from dental biofilm of carious lesions in children aged 6–12 years.

## Results

### Characteristics of foods found at chain supermarkets

**Figure 1:**
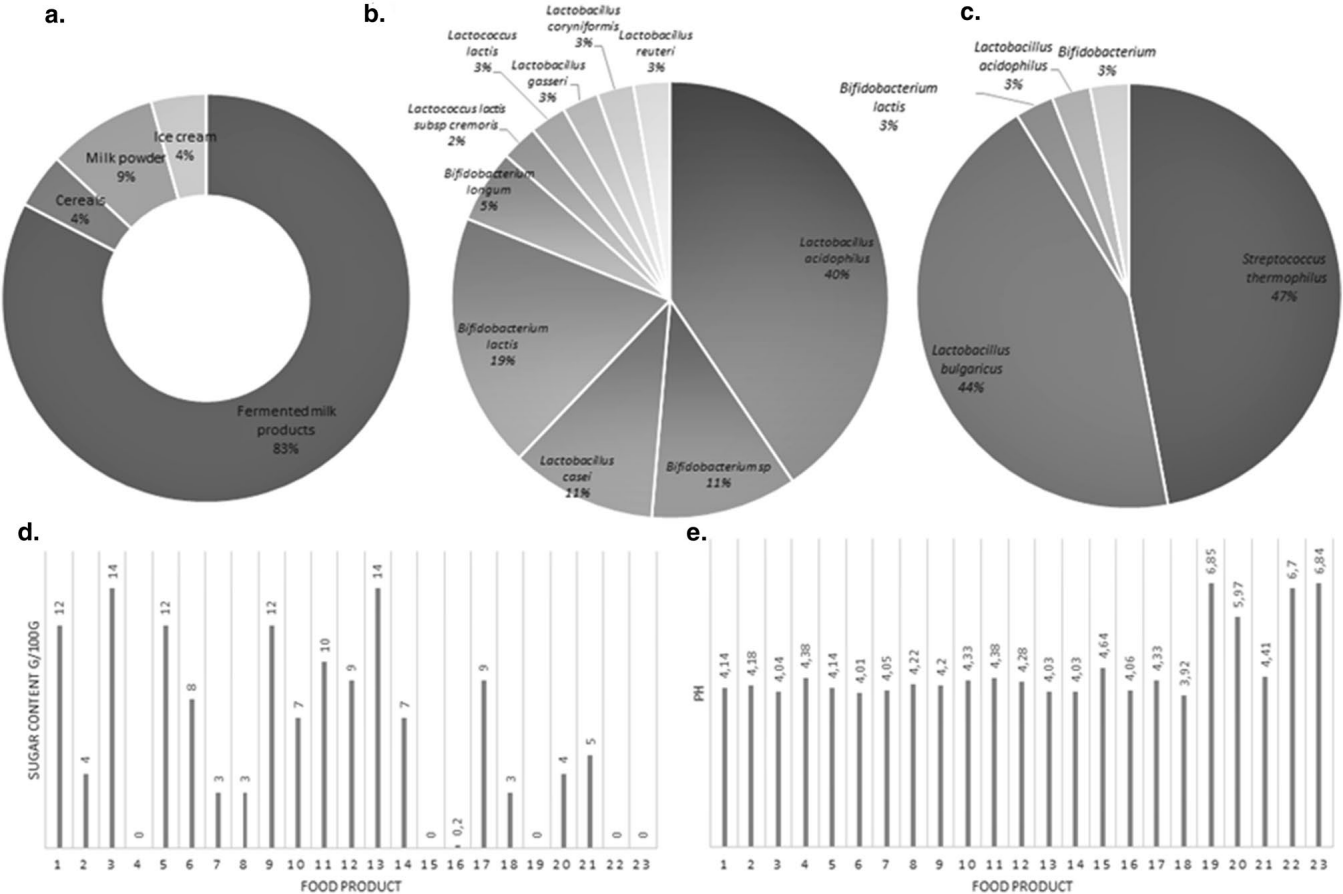
Characteristics of the foods with added probiotics in chain supermarkets in Colombia. (**a**) Type of food. (**b**) Probiotic bacteria announced on food label. (**c**) Fermentative bacteria announced on food label. (**d**) Sugar content announced on food label. (**e**) Initial pH of foods.

In the first phase of the study, 23 functional foods with probiotics were identified from 11 different brands, commercially available in chain supermarkets in the city of Villavicencio (n = 6). The vast majority of these products were fermented milk products (83%), followed by milk powders for babies (9%) (Fig. 1a). The most common probiotic bacteria used were *Lactobacillus* *acidophilus* (40%) followed by *Bifidobacterium* *lactis* (19%) (Fig. 1b) along with the fermentative bacteria, *Streptococcus* *termophilus* (47%) and *Lactobacillus* *bulgaricus* (44%) (Fig. 1c). As for sugar content, 48% of the foods (n = 11) were labeled as having a concentration of more than 5 g/100 g (Fig. 1d) and the initial pH of 83% of the foods was found to be within the range of 3.92–4.64 (Fig. 1e).

### Selection of functional foods with inhibitory capacity against *S*. *mutans* 25175 and a reduced ability to lower the pH of the medium

**Figure 2:**
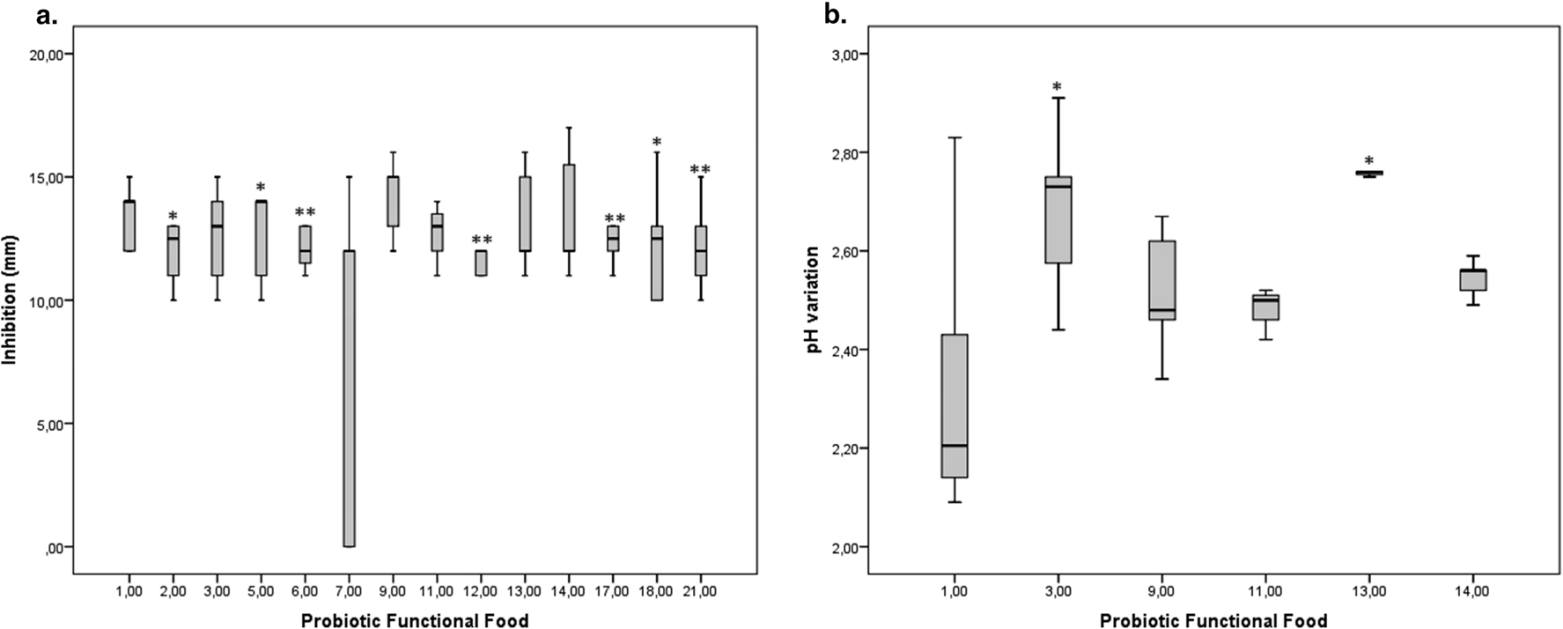
Selection of foods that present the greatest degree of *S*. *mutans* growth inhibition and pH reduction. (**a**) Median inhibition compared with food No. 9 which showed the highest median. (**b**) Foods that presented the highest inhibition compared with food No. 1 which presented the lowest median for pH reduction. Mann–Whitney U test, *P < 0.05, **P < 0.01.

Regarding the inhibition of *S*. *mutans*, it was detected that some of the foods generated a degree of inhibition of ≥ 12 mm (60.9%; n = 14) (Table 1). Food No. 9 presented the largest diameter of inhibition with a median of 15 mm (IQR 13–15), and significant differences were not detected with foods 1, 3, 11, 13 and 14 (P > 0.05) (Fig. 2a). In terms of pH reduction, it was found that most foods caused the pH to lower by more than 2 units (83%, n = 19) (Table 1). Of the foods with a larger inhibition diameter, product No. 1 lowered the pH the least, with a median of 2.20 units (IQR 2.14–2.52), and did not present significant differences (P > 0.05) in terms of the medians of products 9, 11 and 14. On the other hand, significant differences were observed between products 3 and 13, these being foods that lowered the pH of the medium most with 2.73 (2.51–2.76) and 2.75 (2.75–2.76) units respectively (Fig. 2b). Thus, candidates 1, 9, 11 and 14 were selected for the next phase of the study.

**Table 1:** Measurements of *S*. *mutans* inhibition and pH reduction in the culture medium of the different foods analyzed.

Product	Inhibition (mm)		pH decrease	
	Median	IQR	Median	IQR
1	14	12–14	2.20	2.14–2.52
2	12.5	10.5–13	2.27	2.23–2.38
3	13	10.5–14.5	2.73	2.51–2.76
4	10.5	0–11	2.02	1.75–2.35
5	14	11.0–14.0	2.71	2.66–2.73
6	12	11.25–13.0	2.62	2.52–2.76
7	12	0–12	2.28	2.03–2.54
8	0		2.04	1.97–2.19
9	15	13–15	2.48	2.40–2.64
10	11	0–14	2.58	2.53–2.60
11	13	11.5–13.8	2.50	2.42–2.50
12	12	11.0–12.0	2.48	2.46–2.51
13	12	12.0–15.0	2.76	2.75–2.76
14	12	12–15.8	2.56	2.50–2.58
15	10	10–10	2.54	1.98–2.57
16	10	10–10	2.27	2.27–2.28
17	12.5	12.0–13.0	2.61	2.05–2.60
18	12.5	10–13.25	1.88	1.53–2.14
19	0		0.14	0.13–0.14
20	0		0.42	0.12–0.63
21	12	11.0–13.0	1.94	1.81–2.01
22	0		2.23	1.96–2.23
23	0		2.69	2.67–2.69

### Characteristics of the selected foods

Of the selected foods, three were yoghurts (No.1, 11, 14) and one was a fermented milk product that is not categorized as a yoghurt (No. 9). The probiotic bacteria reported to be contained in these foods were *L*. *acidophilus*, *Bifidobacterium* sp., *Bifidobacterium* *animalis* subsp. *lactis* BB12 and *Lactococcus* *casei*. The yoghurts also contained the fermentative bacteria *S*. *thermophilus* and *L*. *bulgaricus*. Food No. 14 had lower sugar content than the other two foods, but no significant differences were found (P > 0.05) from the initial pH of the foods (Table 2).

**Table 2:** Characteristics of the selected foods.

Product	Type of food	Probiotic reported	Fermentative bacteria reported	Sugar content per g/100 g of product	Initial value of pH Mean ± SD	P value Comparison of products’ pH^a^
1	Yoghurt	*Lactobacillus* *acidophilus*, *Bifidobacterium* sp	*S*. *thermophilus*, *L*. *bulgaricus*	12	4.14 ± 0.14	0.22
9	Fermented milk	*Bifidobacterium* *animalis* subsp. *lactis* BB12	*Bifidobacterium* *animalis* subsp. *lactis* BB12	12	4.1 ± 0.15	
11	Yoghurt	*L*. *casei*, *L*. *acidophilus*	*S*. *thermophilus*, *L*. *bulgaricus*	10	4.1 ± 0.09	
14	Yoghurt	*B*. *lactis*, *L*. *acidophilus*	*S*. *thermophilus*, *L*. *bulgaricus*	7	4.04 ± 0.23	

ANOVA test performed according to data normality and homogeneity. *P < 0.05, **P < 0.01.

### Effect of sugar concentration on the inhibition of *S*. *mutans* 25175 and pH reduction

**Figure 3:**
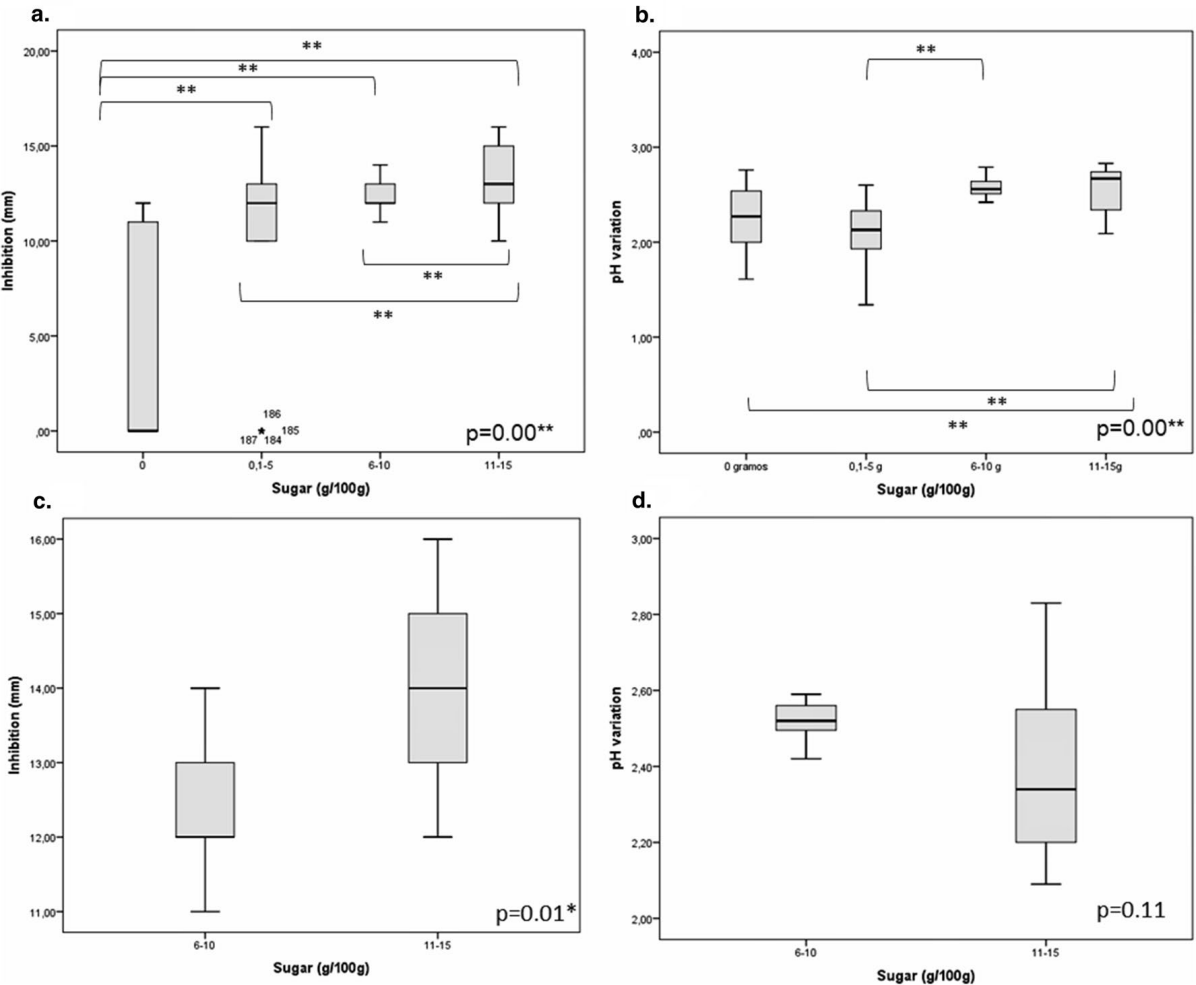
Inhibition of *S*. *mutans* 25175 and pH reduction. (**a**) Relationship between the sugar concentration announced on labels of analyzed foods and *S*. *mutans* 25175 inhibition. (**b**) Relationship between the sugar concentration announced on labels of analyzed foods and the reduction of pH in the medium. (**c**) Relationship between *S*. *mutans* 25175 inhibition and the sugar concentration of the foods selected for the next phase of the study. (**d**) Relationship between the reduction of pH in the medium and the sugar concentration of the foods selected for the next phase of the study. Kruskal–Wallis test and Dunn’s test were used for multiple comparisons, *P < 0.05, **P < 0.01.

By grouping the foods within ranges of sugar concentration (0 g/100 g, 1–5 g/100 g, 6–10 g/100 g and 11–15 g/100 g), it was found that foods with a sugar concentration higher than 10 g/100 g had a significantly greater capacity (P < 0.01) to inhibit *S*. *mutans* 25175 (Fig. 3a). Foods with a sugar concentration of 11–15 g/100 g presented a median inhibition of 13 mm (12–15), compared to unsweetened foods which presented a median of 0 mm (0–11). However, the foods with higher sugar concentrations showed a significant capacity to lower the pH (P < 0.01). Foods with higher sugar concentrations (11–15 g/100 g) displayed a median decrease of 2.67 pH units (2.28–2.74), while unsweetened foods displayed a mean of 2.27 pH units (1.99–2.54) (Fig. 3b). However, according to the sugar range of the selected foods, although the inhibition level was substantial (Fig. 3c), no significant effect on pH lowering results was detected (P > 0.05) (Fig. 3d).

### Analysis of functional foods on mixed cultures of dental biofilm of carious lesions

#### Characteristics of the dental biofilm donors

The majority of the children were male (58%), with an average age of 9.6 ± 1.7. All of the patients had an ICDAS of 2, and 87% had a medium risk of caries (Table 3).

**Table 3:** Characteristics of the population that donated the dental biofilm.

	Frequency (n)	Percentage %
**Sex**		
Male	9	58
Female	6	42
**Age**	Mean: 9.6 ± 1.7	
7	2	13.3
8	2	13.3
9	4	26.7
10	1	6.7
11	4	26.7
12	2	13.3
**ICDAS**		
1	0	0
2	15	100
**Risk of caries**		
Medium	13	86.7
High	2	13.3

#### Effect of selected functional foods on inhibition and lowering of pH in mixed cultures of dental biofilm

This study found that, of the mixed cultures taken from the dental biofilm of carious lesions of 15 children with ICDAS 2, food No. 14 produced the largest inhibition diameter, which was statistically significant (P < 0.01). The inhibition zones produced by this food measured a median of 14 mm (IQR 13–14), followed by products 1 and 9, which produced a statistically similar median inhibition (P > 0.05) of 13 mm (12–13) (Table 4). Despite containing the lowest concentration of sugar among the selected foods (7 g/100 g), food No. 14, was found to lower the pH of the mixed culture combined with the food to similar levels as foods 1 and 9, with a median of 2.83 pH units (2.60–3.0) (P > 0.05).

**Table 4:** Comparison between the inhibition of mixed cultures of dental biofilm from carious lesions and pH reduction of the selected foods.

Product	Inhibition (mm)	
	Median (IQR)	P value^a^
1	13 (12–13)	0.00**
9	13 (12–13)	
11	11 (0–12)	
14	14 (13–14)	

Product	pH decrease	
	Median (IQR)	P value^b^
1	2.83 (2.48–3.01)	0.85
9	2.83 (2.63–3.06)	
11	2.75 (2.54–2.91)	
14	2.83 (2.60–3.0)	

Dunn’s post hoc test for inhibition		
Product 1	Product 2	P value
1	9	0.70
1	11	0.00**
1	14	0.00**
9	11	0.00**
9	14	0.00**
11	14	0.00**

Kruskal–Wallis test and Dunn’s test for multiple comparisons, performed according to data normality and homogeneity. *P < 0.05, **P < 0.01.

#### Comparison between patient sample responses to the selected foods

**Figure 4:**
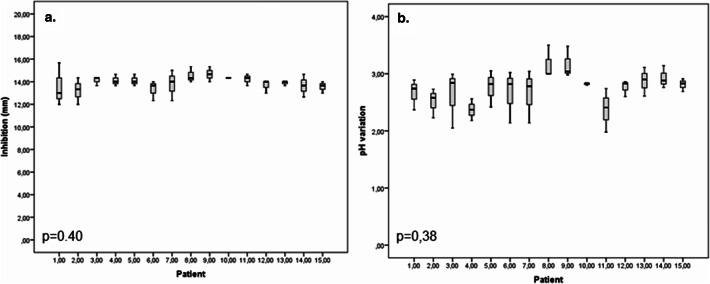
Inhibition ability and the pH reduction of mixed cultures of dental biofilm from carious lesions from 15 patients produced by the foods evaluated. (**a**) Inhibition median produced by product No. 14. (**b**) Median of pH decrease in the mixed culture generated by product No. 14. Kruskal–Wallis test *P < 0.05, **P < 0.01.

No significant differences were found between patient samples (P > 0.05) in terms of the inhibition of the mixed cultures of dental biofilm from carious lesions by functional food No. 14, which produced the largest inhibition diameter (Fig. 4a). Most of the patient samples (80%, n = 15) showed a median inhibition of 14 mm (12–15) for this food, followed by two patient samples which showed a median inhibition of 13 (12.5–14.5).

Regarding the decrease in pH, despite observing a greater variation in the medians produced by the samples taken from the different patients, no significant differences were detected (P > 0.05). The most commonly occurring median decrease among patients was greater than 2.80 (66.7%, n = 10) (2.43–3.52) (Fig. 4b).

## Discussion

This study identified commercially available functional foods with added probiotics that have the capacity to inhibit the growth of *S*. *mutans* 25175 and microorganisms deriving from early carious lesions in children aged 6 to 12. The foods that caused some level of inhibition were the fermented milk products (n = 14), of which three were identified as having a greater ability for inhibition and a lower effect on reducing the pH of the culture medium. The three products included two yoghurts and a fermented milk product that did not meet the categorization for yoghurt^20^. Of these foods, No. 14 displayed the greatest inhibition capacity along with a lower sugar concentration.

Several studies have revealed the efficacy of yoghurts and other fermented milk products in inhibiting the growth of microorganisms associated with caries. The results report a statistically significant decrease in the quantity of *S*. *mutans* in the saliva of patients who have consumed such foods^11–17^. Fermented dairy products have a high potential for caries control, as, in addition to being made up of a consortium of probiotic and/or fermentative bacteria, their metabolites and other antimicrobial substances, they also contain the benefits of milk products such as calcium, phosphate, and lipids, as well as casein phosphopeptides (CPP). These, both contribute to the remineralization of teeth^21^, and inhibit the growth of cariogenic bacteria^22^.

The bacteria announced on the labels of the foods that were the most successful in inhibiting the growth of the mixed culture of bacteria taken from the early carious lesions in children (No. 1, No. 9 and No. 14) included *Lactobacillus* *acidophilus*, *B*. *animalis* subsp. *lactis* BB12, and *Bifidobacterium* sp. Furthermore, *S*. *thermophilus* and *L*. *bulgaricus* were also present as fermentative bacteria in yoghurts. The inhibition caused by the bacteria present in these foods has been attributed to the production of bacteriocins, organic acids, and hydrogen peroxide^23–26^.

Of these bacteria, the most frequently assessed are *B*. *animalis* subsp. *lactis* BB12 and *L*. *acidophilus* which have shown significant inhibition of *S*. *mutans* in the saliva of patients in different age ranges^13,16,17^. The present study found that the food that contained *B*. *animalis* subsp. *lactis* BB12 as its only probiotic and fermentative bacteria (No. 9) presented the greatest inhibition of *S*. *mutans* 25175. However, when evaluating this food with a mixed culture of microorganisms present in the biofilm of carious lesions, its inhibitive capacity was lowered. These results concur with those found in a clinical trial involving 1-month-old newborns, who were given *B*. *animalis* subsp. *lactis* BB12 for almost 2 years, and where, despite the reduction in the numbers of *S*. *mutans*, there was no impact on caries experience^27^. This is probably associated with the vast diversity of microorganisms implicated in the formation of caries, where an inhibition of their growth is not as high as it is with *S*. *mutans*. The authors of this study also concluded that the probiotic did not permanently establish itself in the oral cavity, a result similar to that found in other studies showing that these types of probiotics are transient colonizers^28^.

In relation to lactic acid bacteria such as *S*. *thermophilus* and *L*. *bulgaricus* present in yoghurts, Petti et al.^29^ showed the inhibitory effects of these bacteria on *S*. *mutans* 6519T and other bacteria from the viridans group such as *S*. *oralis* 25671, confirming the important inhibitory properties of these types of bacteria in yoghurt.

Regarding the effect of the foods selected in this study to determine the inhibition of mixed cultures from caries lesions, the yogurt that generated the greatest diameter of inhibition (No. 14) was the one containing a combination of *S*. *thermophilus*, *L*. *bulgaricus*, *L* *acidophilus* and *B*. *lactis*. This can be associated with the synergic action of bacteria present in this food, which potentiate bioactivity such as the foods’ inhibitory capacity against pathogenic microorganisms. This has been demonstrated by studies focusing on the control of other types of diseases^30^.

The importance of studying the effect of the bacteria present in the food on lowering the pH of the culture medium, is given by the fact that considering their lactic acid properties, the release of a high concentration of acids can be counterproductive when supplying them for caries control. The first phase of this study consisted in a selection process, where products No. 3 and 13 were excluded, in spite of presenting good inhibition. These foods significantly lowered the pH of the culture medium (P < 0.05) compared to the other foods selected as candidates for the second phase, probably because they were the foods with the highest sugar concentration (14 g/100 g). This value exceeds the daily sugar concentration recommended by WHO (10% of total calorie intake)^3^.

It is important to point out, at this stage, that of the candidates selected in the first phase (No. 1, 9, 11 and 14), the sugar concentrations of two of the four foods (No. 1 and 9) also exceeded this recommendation, although to a lesser extent (12 g/100 g). A study conducted using the nutritional information of different types of yoghurts commercially available in England found that, although there was evidence that these provided health benefits, most of them had a median quantity of total carbohydrates of over 10 g/100 g, including those developed for children^31^. These types of commercially available foods tend to have high sugar concentrations that could contribute to the appearance of carious lesions in cases where an individual does not apply appropriate diet and hygiene practices.

Thus, commercially available foods that contain sugar concentrations within the limits of low-sugar product categorization and with levels known to have additional health benefits (5 g/100 g) could be considered ideal candidates in terms of supporting oral health. However, in the present study, these types of foods that represented 52% of the evaluated products (n = 12), did not produce a significant inhibition in the growth of *S*. *mutans* 25175. Some of the foods with a sugar content of over 5 g/100 g caused greater inhibition, and therefore demonstrate the role that sugar plays in the biological activity of the bacteria present in fermented milk products^32^. In this respect, and as has been shown in other studies, these types of bacteria cannot be considered ideal as probiotics for use in the oral cavity given the acidification of the medium^33^.

Despite this, it is important to highlight that including these types of bacteria in the diet can contribute to inhibiting the growth of cariogenic microorganisms and therefore complement the effects of other preventative measures for this disease. In fact, the present study identified that three of the four selected candidates (Nos. 1, 9 and 14) inhibited the growth of the mixed culture of microorganisms taken from early carious lesions, and No. 14 produced the highest significant inhibition, had a lower sugar concentration, and reduced the pH to a level similar to the other foodstuffs.

Despite the limitations of this study, such as the lack of tests on biofilm models and a greater diversity of bacteria involved in caries, it was possible to select a functional food supplemented with probiotics that homogeneously inhibited the microorganisms present in the dental plaque of the initial caries lesions of 15 children who attended the dental clinic at three different points in time.

## Conclusion

This study therefore identified a commercially available food with added probiotics suitable for clinical trial. This candidate produced the largest diameter of inhibition for microorganisms present in the biofilm of early carious lesions in 6–12-year-old children and contains less than 10 g/100 g of sugar. Although the bacteria present in these foods are not ideal candidates for oral cavity probiotics, they can help to inhibit the growth of cariogenic microorganisms, and so complement the role of fluoride and good oral health habits. Furthermore, as this food is a milk-based product, it boosts the buffer system of the oral cavity and the remineralization of the tooth enamel.

## Methods

In vitro experimental study carried out in two phases. In the first phase consisted on the selection of a number of commercial foods supplemented with probiotics. These presented a greater inhibitory capacity against the reference strain *S*. *mutans* *ATCC* 25175 and a reduced pH lowering capacity. In the second phase, the inhibition and pH lowering capacity of the selected foods was evaluated against microbial mixed cultures from dental biofilm of children with caries. This study was approved by the subcommittee of ethics (039-2018) at Universidad Cooperativa de Colombia and all procedures were in line with the ethical standards established by the institutional and/or national research committee and with the 1964 Helsinki declaration and its later amendments or comparable ethical standards.

### Selection of functional foods with inhibitory capacity against *S*. *mutans* 25175

Different functional foods supplemented with probiotics available in chain supermarkets in Villavicencio, Colombia, were collected in order to assess only those foods sold in all of Colombia’s cities. The collection criteria were as follows: the foods had to be liquid or dehydrated food for reconstitution with liquid, they had to be suitable for consumption by people of all ages, they had to specify the added probiotics or fermentative bacteria on the packaging, they had to have a sell by date of more than a week, and not be sold in the pharmacy or natural remedies section. These foods were taken to the microbiology laboratory where the information included on their labels was recorded (brand, type of food, probiotic strains, fermentative bacteria, and the quantity of added sugar). Prior to processing, the pH of each one was measured using a pH meter (Accumet AP110, Fisher Scientific, Pittsburgh, USA) with a microelectrode (FisherbrandTM AccumetTM Micro Glass Mercury-Free Combination Electrode, Fisher Scientific, Pittsburgh, USA).

For the inhibition test, 20 mL of the solid Brain Heart Infusion (BHI) (Oxoid) was inoculated with the strain *S*. *mutans* ATCC 25175 (Microbiologics, Minnesota, USA), using the spread plate technique, in a concentration of 1–2 × 10^8^ UFC/mL^18^. Then, holes were bored in the medium, creating 9 mm diameter wells for the inoculation of 100 µL of the foodstuffs. Clorhexidina at 0.2% (positive control) and a sterile saline solution at 0.9% (negative control) were used as controls for the inhibition. The cultures were incubated at 37 °C with 5% CO_2_ over 15 h to determine whether inhibition had taken place. If so, the diameter of the zone of inhibition or halo was measured. To determine acid production and therefore the pH reduction generated by the bacteria present in the functional foods, 100 µL of the foods were inoculated in BHI broth (Oxoid) and the pH was measured before and after incubation at 37 °C with 5% CO_2_ over 15 h. Inhibition and pH lowering tests were performed in triplicate on different days using 3 different lots in Villavicencio and 2 in Medellín, Colombia. According to the results of this study, foods were selected for the next phase, taking into account those that presented greater inhibition of *S*. *mutans* and lower acid production.

### Analysis of selected foods in relation to mixed cultures of carious lesions in children aged 6–12 years

For this phase, parents or caregivers of children aged 6–12 years authorized their children's participation in the study, by means of a signed written informed consent, while the children agreed to participate by giving their written assent. They were interviewed by a pediatric dentist calibrated in ICDAS (kappa value ≥ 0.7) in order to find the child's clinical history, and subsequently examine and define whether they fell in line with the selection criteria. The criteria included having ICDAS 1 and 2 caries lesions, and moderate or high caries risk according to individual caries risk assessment using a *riesgograma*^19^, which determines 7 risk factors: history of caries, the presence of dental biofilm, local/systemic factors of plaque retention, content of the diet, frequency of eating during the day, use of fluoride and reason for last dental appointment. Furthermore, the patients had to be free from systemic illnesses; have no orthodontic appliances fitted; should not have taken antibiotics in the last 3 months; and, at the time of sample taking, should not have brushed their teeth in the 8 h prior. For the microbiological analysis, dental plaque was taken from the lesion using a microspoon until filling an inoculation loop calibrated to 2 µL. The samples were deposited independently in Eppendorf tubes with glass pearls and 1,000 µL of saline solution, and immediately transported to the laboratory.

### Bacteria inhibition and pH reduction analysis

For the inhibition tests, the samples of dental plaque were mixed by vortex mixer (V1 Plus, BOECO, Hamburg, Germany) for 1 min. Then, 100 µL of the dental plaque was inoculated in 20 mL of solid BHI (Oxoid) using the spread plate technique. The holes had a diameter of 9 mm where 100 µL of the selected foods and controls were deposited. After incubating at 37 °C for 15 h with 5% CO_2_, the inhibition and size of the halo was determined. For the pH reduction analysis, the pH was determined before and after incubating 100 µL of the dental biofilm sample and 100 µL of the foodstuff in 1,000 µL of BHI broth. These analyses were carried out using samples taken from a lesion from the same child on three different days and in triplicate. The foods originated from three distinct lots.

### Statistical analysis

The SPSS version 25 (IBM Corp, Armonk, NY, USA) software was used to conduct the analyses. Data normality was determined using the Kolmogorov–Smirnov test and variance homogeneity using the Levene test. The Kruskal–Wallis test was used to determine whether there were differences in terms of inhibition and pH reduction generated by the bacteria present in the different foods. To define the foods with the best response, the results were compared with the food with the highest inhibition level and with the food with the lowest degree of pH reduction, using the U Mann–Whitney test.

To analyze the microorganism inhibition and pH reduction capacity of selected foods in mixed cultures of lesions with caries, the results pertaining to selected foods were examined using the Kruskal–Wallis test and compared using Dunn's post-hoc test.
